# Application of a whole blood mycobacterial growth inhibition assay to study immunity against *Mycobacterium tuberculosis* in a high tuberculosis burden population

**DOI:** 10.1371/journal.pone.0184563

**Published:** 2017-09-08

**Authors:** Richard Baguma, Adam Penn-Nicholson, Erica Smit, Mzwandile Erasmus, Jonathan Day, Lebohang Makhethe, Marwou de Kock, E. Jane Hughes, Michele van Rooyen, Bernadette Pienaar, Lynnett Stone, Willem Hanekom, Michael J. Brennan, Robert S. Wallis, Mark Hatherill, Thomas J. Scriba

**Affiliations:** 1 South African Tuberculosis Vaccine Initiative (SATVI), Institute of Infectious Disease and Molecular Medicine and Division of Immunology, Department of Pathology, University of Cape Town, Cape Town, South Africa; 2 Aeras, Rockville, Maryland, United States of America; 3 The Aurum Institute, Johannesburg, South Africa; Fundació Institut d’Investigació en Ciències de la Salut Germans Trias i Pujol, Universitat Autònoma de Barcelona, SPAIN

## Abstract

The determinants of immunological protection against *Mycobacterium tuberculosis (M*.*tb)* infection in humans are not known. Mycobacterial growth inhibition assays have potential utility as *in vitro* surrogates of *in vivo* immunological control of *M*.*tb*. We evaluated a whole blood growth inhibition assay in a setting with high burden of TB and aimed to identify immune responses that correlate with control of mycobacterial growth. We hypothesized that individuals with underlying *M*.*tb* infection will exhibit greater *M*.*tb* growth inhibition than uninfected individuals and that children aged 4 to 12 years, an age during which TB incidence is curiously low, will also exhibit greater *M*.*tb* growth inhibition than adolescents or adults. Neither *M*.*tb* infection status, age of the study participants, nor *M*.*tb* strain was associated with differential control of mycobacterial growth. Abundance and function of innate or T cell responses were also not associated with mycobacterial growth. Our data suggest that this assay does not provide a useful measure of age-associated differential host control of *M*.*tb* infection in a high TB burden setting. We propose that universally high levels of mycobacterial sensitization (through environmental non-tuberculous mycobacteria and/or universal BCG vaccination) in persons from high TB burden settings may impart broad inhibition of mycobacterial growth, irrespective of *M*.*tb* infection status. This sensitization may mask the augmentative effects of mycobacterial sensitization on *M*.*tb* growth inhibition that is typical in low burden settings.

## Introduction

Almost a quarter of the world’s population is estimated to be infected with *Mycobacterium tuberculosis (M*.*tb)* [1], driving the most deadly epidemic due to an infectious agent. In 2015, approximately 10.4 million people developed active tuberculosis (TB) disease, resulting in 1.4 million deaths [2]. Several risk factors for developing TB disease following *M*.*tb* infection have been identified, including compromised immunity due to HIV co-infection, diabetes, gender and age [3]. Pre-adolescent children older than 4 years of age have much lower rates of progression to TB disease following infection than adolescents or adults [4–7]. Furthermore, in such children pulmonary TB typically manifests as a mild, pauci-bacillary disease, whereas adolescents and adults more commonly present with multi-bacillary disease, more pronounced lung infiltration with immunopathology and lung cavitation [6,7]. The low risk of TB in children within this “golden age of TB” presents an opportunity to study natural resistance and/or characteristics of successful immunity to *M*.*tb* in humans, which are not well understood.

The need for a TB vaccine that is more efficacious than Bacillus Calmette-Guérin (BCG) is urgent. Efforts to develop TB vaccines are hampered by the lack of reliable immunological correlates of protection or biomarkers that predict vaccine efficacy [8]. The adaptive immune response mediated by T cells is necessary for host control of *M*.*tb* infection [9]. As a consequence, frequencies of antigen-specific IFN-γ-producing and polyfunctional CD4 T cells co-expressing IFN-γ, TNF-α and IL-2 have been commonly measured markers of vaccine immunogenicity in preclinical and clinical testing of TB vaccine candidates. However, antigen-specific Th1 cells are not sufficient for complete protection in mouse models of TB [10,11]. In human infants, frequencies and cytokine co-expression profiles of BCG-specific CD4 and CD8 T cells did not correlate with risk of TB [12,13].

Efforts to screen and select the most promising novel vaccine candidates ideally require a functional assay that can measure immune-mediated control or killing of intracellular mycobacteria. A number of mycobacterial growth inhibition assays (MGIA) have been developed to measure functional inhibition of mycobacterial replication by blood leukocytes [14–21]. Animal studies in mice [22,23] and cattle [24] have shown that *in vitro* mycobacterial growth inhibition corresponded with control of *in vivo* mycobacterial replication, highlighting the potential utility of MGIAs as correlates of protection.

In humans, significantly lower mycobacterial growth in whole blood from BCG vaccinated individuals or those previously infected with *M*.*tb*, compared with blood from individuals not sensitized to mycobacteria, have been reported [17,19,20]. These studies were conducted with very small numbers of participants from settings with very low TB incidence. It is not known to which degree mycobacterial growth inhibition would be influenced by much higher levels of mycobacterial sensitization, as is typical in settings with high TB burden.

Here, we aimed to evaluate the utility of a whole blood based MGIA in a setting with a very high burden of TB and to identify functional and phenotypic characteristics of peripheral blood immune responses that correlate with mycobacterial growth inhibition.

We hypothesized that individuals with underlying *M*.*tb* infection or mycobacterial sensitization will exhibit greater *M*.*tb* growth inhibition than uninfected individuals. We also hypothesized that children in the golden age of TB, between 4 and 12 years, will exhibit greater *M*.*tb* growth inhibition than adolescents or adults.

## Materials and methods

### Ethics statement

This study was approved by the University of Cape Town Human Research Ethics Committee. Written informed consent was obtained from adults prior to enrollment. Children provided written informed assent while their legal guardians provided written informed consent prior to enrollment.

### Study design and participants

This was a cross-sectional study comprising three cohorts of healthy, HIV-negative participants from the Worcester region in the Western Cape Province of South Africa, a setting with high burden of TB. Individuals with any acute or chronic disease, those taking immunos^#ressive medication, with a previous diagnosis of active TB disease or who currently or previously participated in a TB vaccine trial, were excluded. Women who were pregnant or lactating were also excluded from participation. We aimed to enroll adults aged 19 to 51 years into the 1^st^ cohort; young adults aged 18 years into the 2^nd^ cohort; and 8 year old children into a 3^rd^ cohort, with equal numbers of *M*.*tb*-infected and uninfected participants into each cohort. *M*.*tb*-infection status was assessed using the QuantiFERON-TB Gold In-Tube Assay (QFT) (QIAgen), according to the manufacturer’s instructions. HIV infection was diagnosed by rapid HIV-antibody test. Some aspects of the adult cohort have been described before [25].

### Mycobacterial strains

*Mycobacterium bovis* BCG (Danish 1331, Statens Serum Institute), *M*.*tb* H37Rv (donated by Christopher Sassetti, University of Massachusetts), *M*.*tb* HN878 and *M*.*tb* CDC1551 were grown in Middlebrook 7H9 liquid medium (Sigma-Aldrich). For each batch of mycobacterial stock, the mycobacterial inoculation volume was established by titration to achieve a time-to-positivity (TTP) of 6.5 days in mycobacterial growth indicator tubes (MGIT) in a BACTEC MGIT 960 machine (BD Biosciences), using a 3-parameter exponential decay curve, as previously optimized for immunological MGIA assays [19]. These volumes equated to inocula between 8.5x10^3^ colony forming units [CFU]/mL and 2.4x10^4^ CFU/mL.

### Blood sample collection and whole blood mycobacterial growth inhibition assay

Venous blood was collected from participants directly into QFT tubes, and into sodium heparin tubes to be used for mycobacterial growth inhibition assays, whole blood stimulations and flow cytometry analysis.

MGIA were performed in duplicate in Sarstedt tubes containing 300μl fresh whole blood and 300μl RPMI, inoculated with each individual mycobacterial strain (average TTP standard deviation of 0.09 days). Tubes were incubated for 96 hours at 37°C with slow constant rotation. The host cells in each duplicate tube were then lysed with 1mL sterile water and bacilli sedimented at 15,300 x g for 10 minutes before being resuspended in 500μl Middlebrook 7H9 broth and the entire volume inoculated into BACTEC MGIT (MGIT, all from Becton Dickinson). MGIT tubes were incubated in a BACTEC MGIT 960 until growth was detected. For each run, a “direct-to-MGIT” positive control, comprising direct mycobacterial inoculation of MGIT tubes, and incubated in parallel with the blood was performed to control for minor variability in inoculum. The log change in viability in each whole blood culture was calculated as log (final)–log (initial), where initial and final are the apparent volumes of the inoculum and the completed culture based on their respective TTP values, calculated using the titration curve. Results are reported as log change per day of whole blood culture. Data management, standard curves, interpolations, and calculation of change in mycobacterial growth were done using an Excel macro provided by one of the co-authors (R.W), as previously described [21].

### Whole blood intracellular cytokine staining assays

Whole blood intracellular cytokine staining (ICS) assays to measure T cell and innate responses were performed using a qualified assay as previously described [25–27], with slight modifications. Briefly, 0.5 mL of heparinized whole blood was stimulated with antigens (see below) at 37°C for either 7 or 3 hours (see below). Brefeldin A (10 μg/mL; Sigma-Aldrich) was then added and the blood further incubated for another 5 or 3 hours. Stimulated whole blood was treated with 2 mM EDTA (Sigma-Aldrich), red blood cells were then lysed and white blood cells fixed using FACS Lysing solution (BD Biosciences). Fixed cells were either stained for immediate flow cytometric analysis or cryopreserved for batch analysis. TruCount beads (BD Biosciences) were included to determine the absolute number of each cell subset per milliliter of whole blood.

#### 12 hr T cell whole blood assay

Blood was incubated in the presence of co-stimulatory antibodies, anti-CD28 and anti-CD49d (at 1 μg/mL each; BD Biosciences, San Jose, CA) at 37°C for 7 hours with either lyophilized BCG Danish 1331 (1.2 X10^6^ CFU/mL, resuspended from the vaccine vial in RMPI; Statens Serum Institute), 15-mer peptide pool of ESAT-6 and CFP-10 overlapping by 10 amino acids (1 μg/mL) or phytohemagglutinin (PHA; 5 μg/mL; Sigma-Aldrich) or was left unstimulated. Brefeldin A was then added, and the blood was incubated for a further 5 hours.

#### 6 hr innate cell whole blood assay

Blood was incubated at 37°C for 3 hours with either lyophilized BCG (Danish strain 1331; 2 X10^6^ colony forming units [CFU]/mL) or recombinant BCG expressing green fluorescent protein (BCG-GFP, Pasteur strain; 3.5 X10^5^ colony forming units [CFU]/mL; donated by Muazzam Jacobs, University of Cape Town), Lipopolysaccharide (LPS; 100 ng/mL; Sigma-Aldrich) or was left unstimulated. Brefeldin A was then added, and the blood was incubated for a further 3 hours.

### Whole blood intracellular cytokine staining (ICS) and flow cytometry

The following monoclonal antibodies were used for surface and intracellular staining for flow cytometry.

#### T cell panel

anti-CD3-BV421 (300434, from Biolegend), anti-CD4-QDot605 (Q10008, from Life Technologies), anti-CD8-PerCP-Cy5.5 (341050), anti-γδTCR-APC (55718), anti-IFN-γ-AF700 (557995), anti-IL-2-FITC (340448) all from BD Biosciences, anti-TNFα-PE-Cy7 (25-7349-82) and anti-IL-17 (CAP17)-PE (12–7178) both from eBiosciences.

#### Innate cell panel

anti-CD14-BV570 (301831, Biolegend), anti-CD11c-PerCP-Cy5.5 (337210), anti-HLA-DR-AlexaFlour700 (307626) all from Biolegend, anti-CD66a/c/e-QDot605 (342302, Biolegend, with in-house conjugation to QDot605, Q-22001), anti-CD16-PE-Cy5 (555408, from BD Biosciences), anti-IL-12-PE (554575), anti-TNF-α-PE-Cy7 (25-7349-82) both from eBioscience, anti-IL-10-PE (506804), anti-IL-6-APC (501112), anti-CD3-BV421 (300434), anti-CD19-BV421 (302233) and anti-CD335-BV421 (331913) all from Biolegend.

Cryopreserved, fixed white cells were washed in PBS and stained for 1 hour at 4°C with surface marker antibodies. For intracellular staining, cells were permeabilized with Perm/Wash buffer (BD Biosciences) and incubated for 1 hour at 4°C with antibodies for intracellular markers. Fixed cells were then acquired on a LSR II flow cytometer (BD Biosciences).

### Data analysis

Flow cytometry data was analyzed using FlowJo software (v9.7.2; Tree Star). When appropriate, background subtraction for ICS data was performed in Pestle, and data sorting and visualization was done in SPICE [28]. Statistical analyses and graphing were performed using PRISM (GraphPad Software v6, San Diego, Calif.). Nonparametric unpaired statistical analysis (Mann-Whitney U test) was used to determine differences between participant groups. Spearman’s rank correlation coefficients were calculated for correlation analyses. A p-value of less than 0.05 was considered significant.

## Results

### Study participants

In total, 161 participants were enrolled into the three cohorts of this study, 55 adults aged 19 to 51 years, 58 young adults aged 18 years and 48 children aged 8 years. Participants in each age group were approximately equally stratified according to *M*.*tb* infection status by QuantiFERON, which shows excellent agreement with TST in this setting [29,30]. All participants had a history of BCG vaccination. The demographic characteristics of the study participants are summarized in Table 1.

**Table 1:** Demographic characteristics of enrolled participants.

	Adults (19–51 years)		Young adults		Children	
	QFT+	QFT-	QFT+	QFT-	QFT+	QFT-
**Participants, n**	29	26	37	21	20	28
**Female, n (%)**	19 (66)	19 (73)	21 (57)	10 (48)	9 (45)	15 (54)
**Mean age, years**	30	32	18	18	8	8
**Ethnicity, n (%)**						
** Black African**	10 (35)	4 (14)	17 (46)	6 (28)	0 (0)	3 (11)
** Mixed race**	17 (59)	13 (50)	20 (54)	14 (67)	20 (100)	25 (89)
** Caucasian**	1 (3)	9 (36)	0 (0)	1 (5)	0 (0)	0 (0)
** Indian**	1 (3)	0 (0)	0 (0)	0 (0)	0 (0)	0 (0)

### Influence of *M*.*tb* infection status on mycobacterial growth

**Fig 1:**
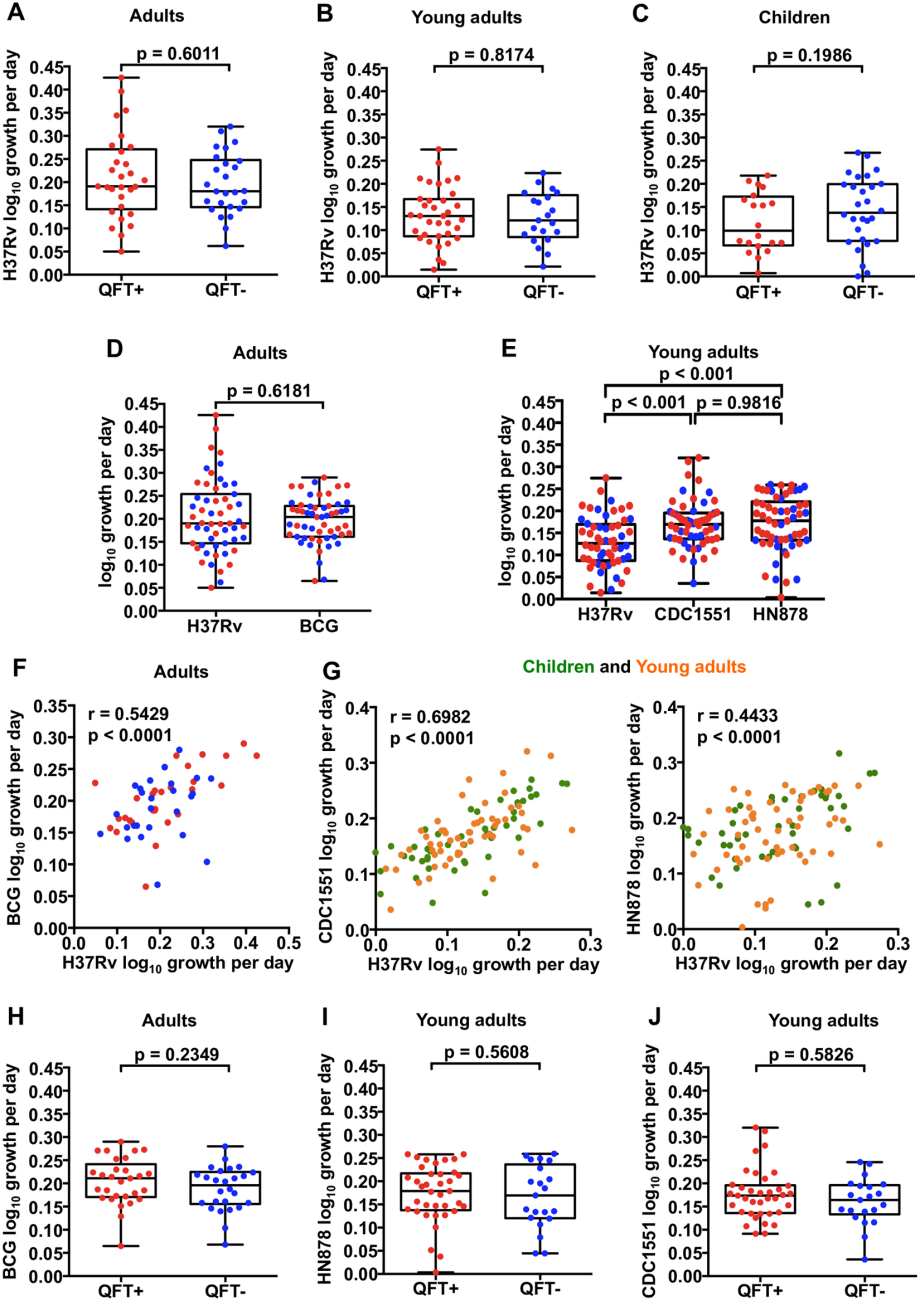
*In vitro* mycobacterial growth inhibition in *M*.*tb*-infected (QFT+) and uninfected (QFT-) individuals. Inhibition of *M*.*tb* H37Rv growth by whole blood from QFT+ and QFT- adults (**A**), young adults (**B**) and children (**C**) assessed using a mycobacterial growth inhibition assay (MGIA). The growths of *M*. *bovis* BCG, *M*.*tb* H37Rv, *M*.*tb* isolate CDC1551 and *M*.*tb* isolate HN878 in the whole blood of adults (**D**) and young adults (**E**), respectively were compared. Spearman’s correlation analyses of the growth of different mycobacterial strains (**F** and **G**). The growth of BCG (**H**), HN878 and CDC1551 (**I** and **J**), was measured in the whole blood of adults and young adults, respectively. The red and blue circles represent QFT+ and QFT- individuals, respectively while the green and orange circles represent children and young adults respectively. The horizontal line represents the median, the boxes represent the interquartile range, and the whiskers represent the range. Differences in mycobacterial growth inhibition between both groups of individuals were evaluated with the Mann-Whitney test (shown *P* values).

We sought to assess the utility of the whole blood MGIA to measure functional inhibition of mycobacterial replication by blood leukocytes in healthy individuals with or without *M*.*tb* infection. Since individuals with *M*.*tb* infection typically have higher, more activated and/or differentiated mycobacteria-specific T cell responses [25], we hypothesized that QFT+ individuals would control the growth of *M*.*tb* to a greater degree than uninfected individuals. However, no significant difference in *M*.*tb* H37Rv growth was observed between 26 QFT- and 29 QFT+ adults (Fig 1A). Similarly, *M*.*tb* H37Rv growth was also not different between the 21 QFT- and 37 QFT+ young adults (Fig 1B) or 28 QFT- and 20 QFT+ children (Fig 1C), and comparable to previous results in a non-endemic setting [21].

We also explored if the magnitude of the *M*.*tb*-specific IFN-γ response measured by the QFT assay, a test for ESAT-6- and CFP-10-specific CD4 T cells [25], was associated with mycobacterial growth *in vitro*. No correlation between IFN-γ QFT response (a marker of *M*.*tb* infection) and inhibition of *M*.*tb* H37Rv growth was observed (S1 Fig).

### Growth inhibition of different mycobacterial strains

To determine the importance of the inoculum strain in outcome of the mycobacterial growth inhibition assay, we performed parallel MGIAs using *M*.*tb* H37Rv and *Mycobacterium bovis* BCG in the adult cohort, or parallel assays using H37Rv, the virulent W/Beijing *M*.*tb* strain HN878, and the less virulent Euro-American *M*.*tb* strain, CDC1551. We hypothesized that growth of BCG, and *M*.*tb* CDC1551 would be inhibited to a greater degree than the virulent *M*.*tb* HN878 [31,32]. In the adult cohort, growth of H37Rv and BCG were not different (Fig 1D). In young adults, growth of H37Rv was significantly lower compared to CDC1551 and HN878 (Fig 1E). Nevertheless, growth of H37Rv correlated significantly with that of CDC1551 (r = 0.6982, p < 0.0001) and HN878 (r = 0.4433, p < 0.0001) in young adults and children (Fig 1G), and with BCG in adults (r = 0.5429, p < 0.0001) (Fig 1F). In QFT- and QFT+ participants, growth of BCG was similar in adults (Fig 1H), as was growth of HN878 and CDC1551 in young adults (Fig 1I and 1J). These data suggest that the inoculum strain is not a major determinant in mycobacterial growth inhibition assays.

### Influence of age on mycobacterial growth

**Fig 2:**
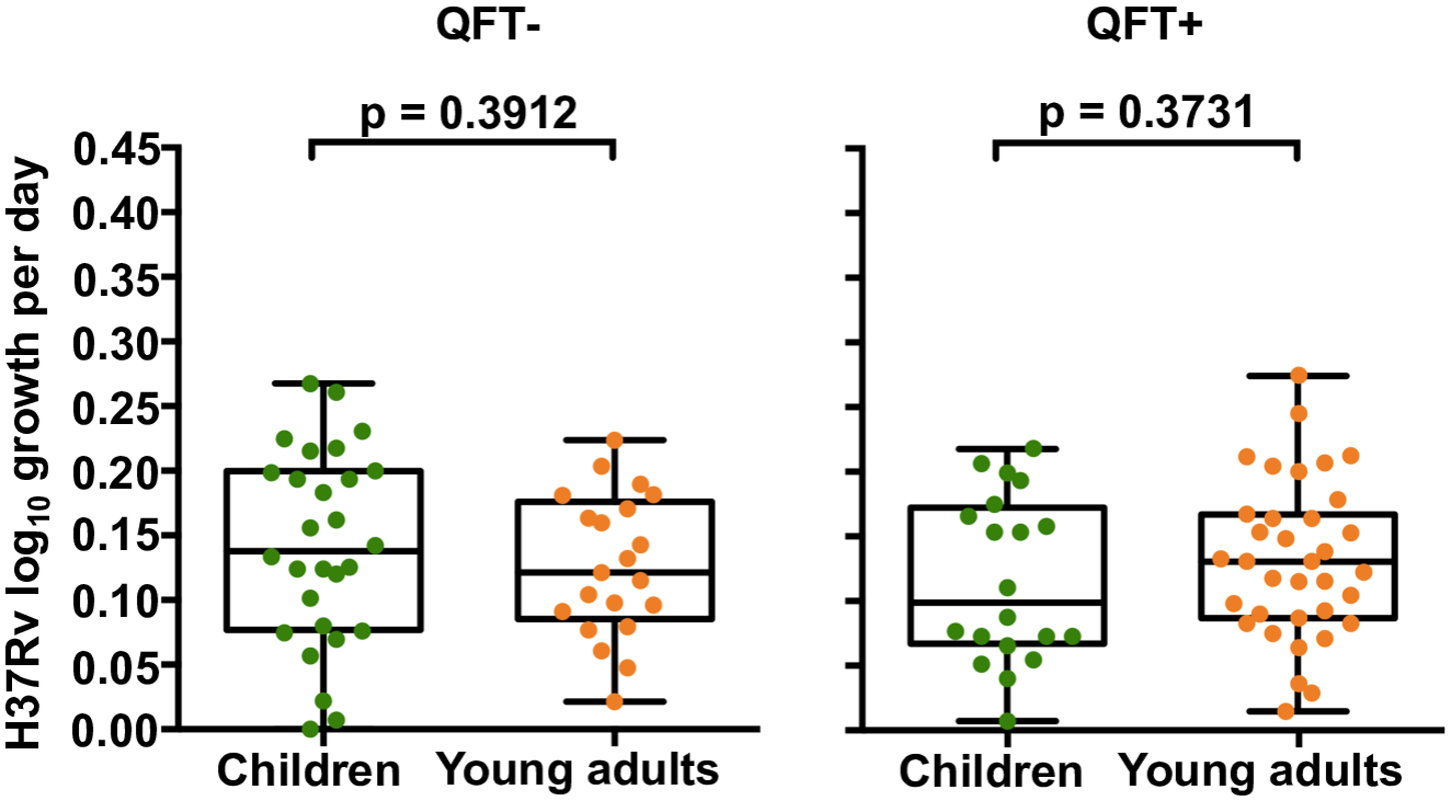
*In vitro* mycobacterial growth inhibition in children and young adults. Inhibition of *M*.*tb* H37Rv growth by whole blood from QFT- and QFT+ 8 and 18 year olds. Green and orange circles represent children and young adults, respectively. The horizontal line represents the median, the boxes represent the interquartile range, and the whiskers represent the range. Differences in mycobacterial growth inhibition between both groups of individuals were evaluated with the Mann-Whitney test (shown *P* values).

Next, we aimed to determine if inhibition of mycobacterial growth is different in children aged between 4 and 12 years, compared with young adults. In light of the low risk of TB disease in children within the Golden Age, we hypothesized that *in vitro* inhibition of *M*.*tb* H37Rv growth would be greater in 8 year old children compared to 18 year old young adults. However, no difference in *M*.*tb* H37Rv growth was observed between whole blood cultures from QFT- or QFT+ 8 and 18 year olds (Fig 2). *M*.*tb* CDC1551 and HN878 growth were also not different between QFT- 8 and 18 year olds (p = 0.413 and p = 0.328, respectively) and QFT+ 8 and 18 year olds (p = 0.260 and p = 0.297, respectively; data not shown).

### Innate cell function and control of mycobacterial growth

**Fig 3:**
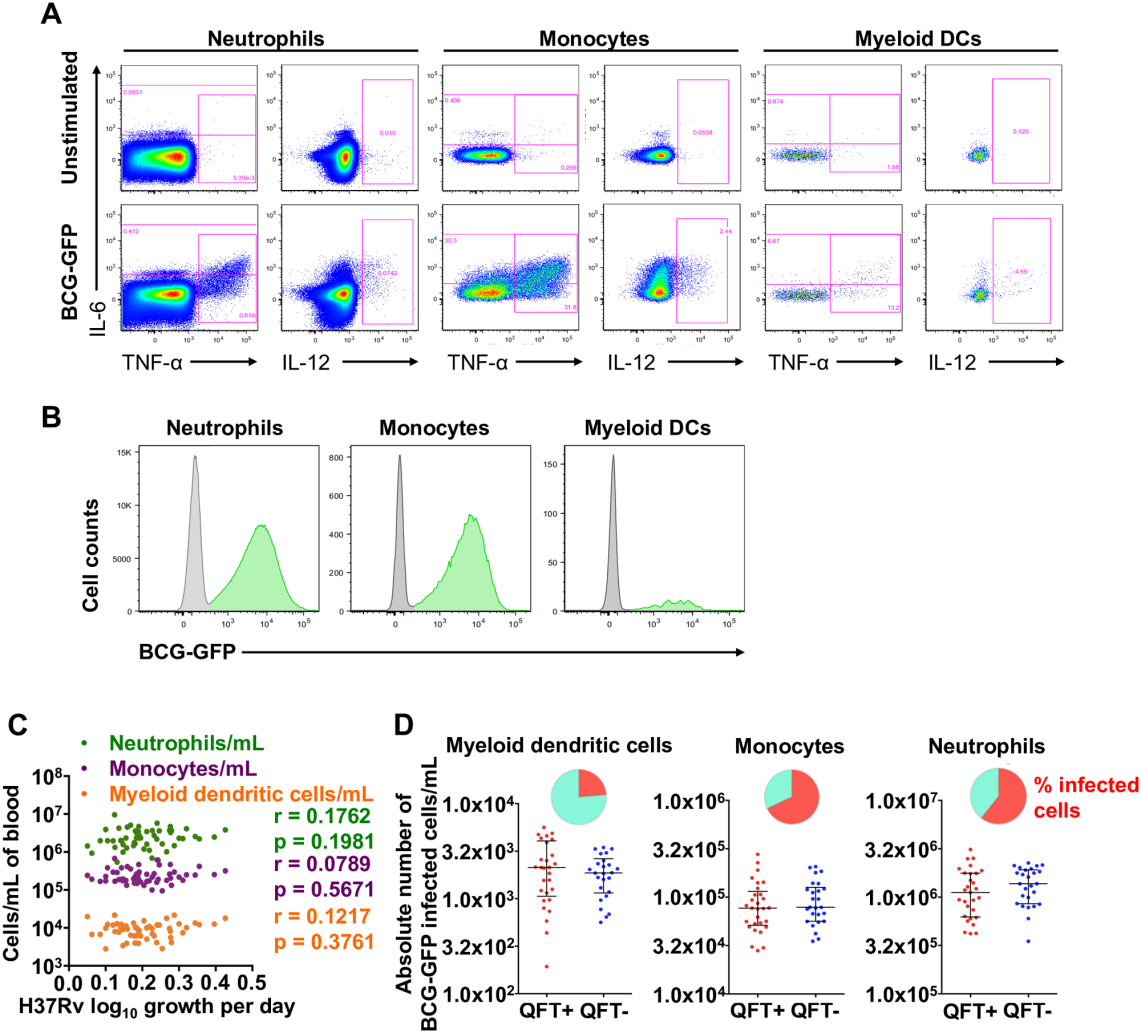
Detection of GFP-expressing BCG by innate cells and association between absolute numbers of innate cells and mycobacterial growth inhibition. (**A**) Representative flow cytometry plot of IL-6, IL-12 and TNF-α cytokine expression by myeloid dentritic cells (mDCs), monocytes and neutrophils, measured in whole blood stimulated for 6 hours with BCG, BCG-GFP (shown) or LPS, relative to an unstimulated control sample. (**B**) Representative histograms indicating proportions of innate cells that phagocytosed BCG-GFP (green). (**C**) Absolute numbers of innate cell subsets per milliliter of unstimulated whole blood plotted against *M*.*tb* H37Rv growth. R and p values were calculated using Spearman’s correlation. (**D**) Absolute numbers of BCG-GFP-positive mDCs, monocytes or neutrophils per mL of whole blood in adult individuals, stratified by QFT status. The inclusion of TruCount beads during the cell staining steps of the innate whole blood assay allowed determination of the absolute number of each subset of cells per mL of whole blood. The red and blue circles represent QFT+ and QFT- adults, respectively. Horizontal lines represent medians and whiskers, the interquartile range. Differences in absolute counts of BCG-GFP-positive innate cells between the groups were evaluated with the Mann-Whitney test (shown *P* values). The pie charts show relative proportions of BCG-GFP-positive cells among each innate cell subset.

We hypothesized that whole blood growth inhibition would be mediated by innate cell responses to mycobacteria and sought to determine if frequencies and functions of innate cells were associated with mycobacterial growth. Neutrophil, monocyte and myeloid dendritic cell (mDC) numbers were enumerated, uptake of green fluorescent protein (GFP)-expressing BCG was measured and intracellular cytokine expression was measured by flow cytometry (Fig 3 and S2A Fig). No association was found between absolute counts of neutrophils, monocytes or myeloid dendritic cells and *M*.*tb* H37Rv growth in whole blood from adult participants (Fig 3C). Similarly, neither absolute counts nor relative proportions of GFP-positive innate cells were different between QFT+ and QFT- adults (Fig 3D) and did not correlate with *M*.*tb* H37Rv growth inhibition in whole blood (data not shown).

**Fig 4:**
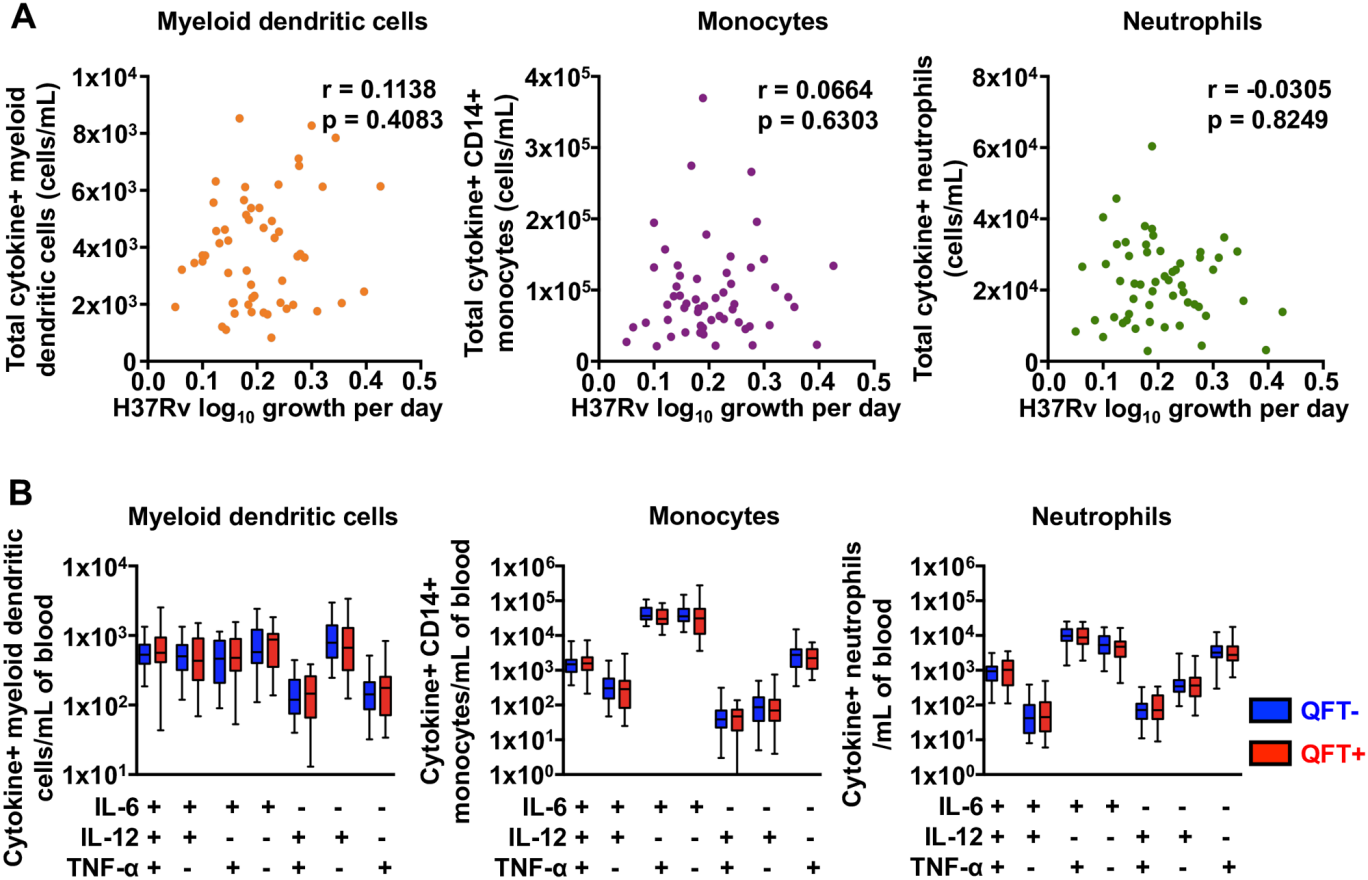
Mycobacteria-specific cytokine expression by innate cells in whole blood from adults and mycobacterial growth inhibition. (**A**) Absolute numbers of total cytokine expressing innate cells per mL of whole blood plotted against *M*.*tb* H37Rv growth. R and p values were calculated using Spearman’s correlation analysis. (**B**) Numbers of BCG-specific mDCs, monocytes and neutrophils co-expressing IL-6, IL-12 and/or TNF-α in whole blood from QFT+ (red) and QFT- (blue) adults. Medians are represented by the horizontal line, interquartile ranges by the boxes, and ranges by the whiskers. The Mann-Whitney test was used to assess differences between QFT+ and QFT- adults and none were found to be different.

Finally, we determined if absolute counts of BCG-GFP-specific innate cells co-expressing IL-6, IL-12 and/or TNF-α were associated with control of *M*.*tb* growth. Again, no correlation between absolute numbers of total cytokine expressing innate cells and control of mycobacterial growth was observed (Fig 4A). Further, there was no difference in numbers of mDCs, monocytes and neutrophils expressing specific patterns of innate cytokines between QFT+ and QFT- adults (Fig 4B).

### Antigen-specific T cell function and control of mycobacterial growth

**Fig 5:**
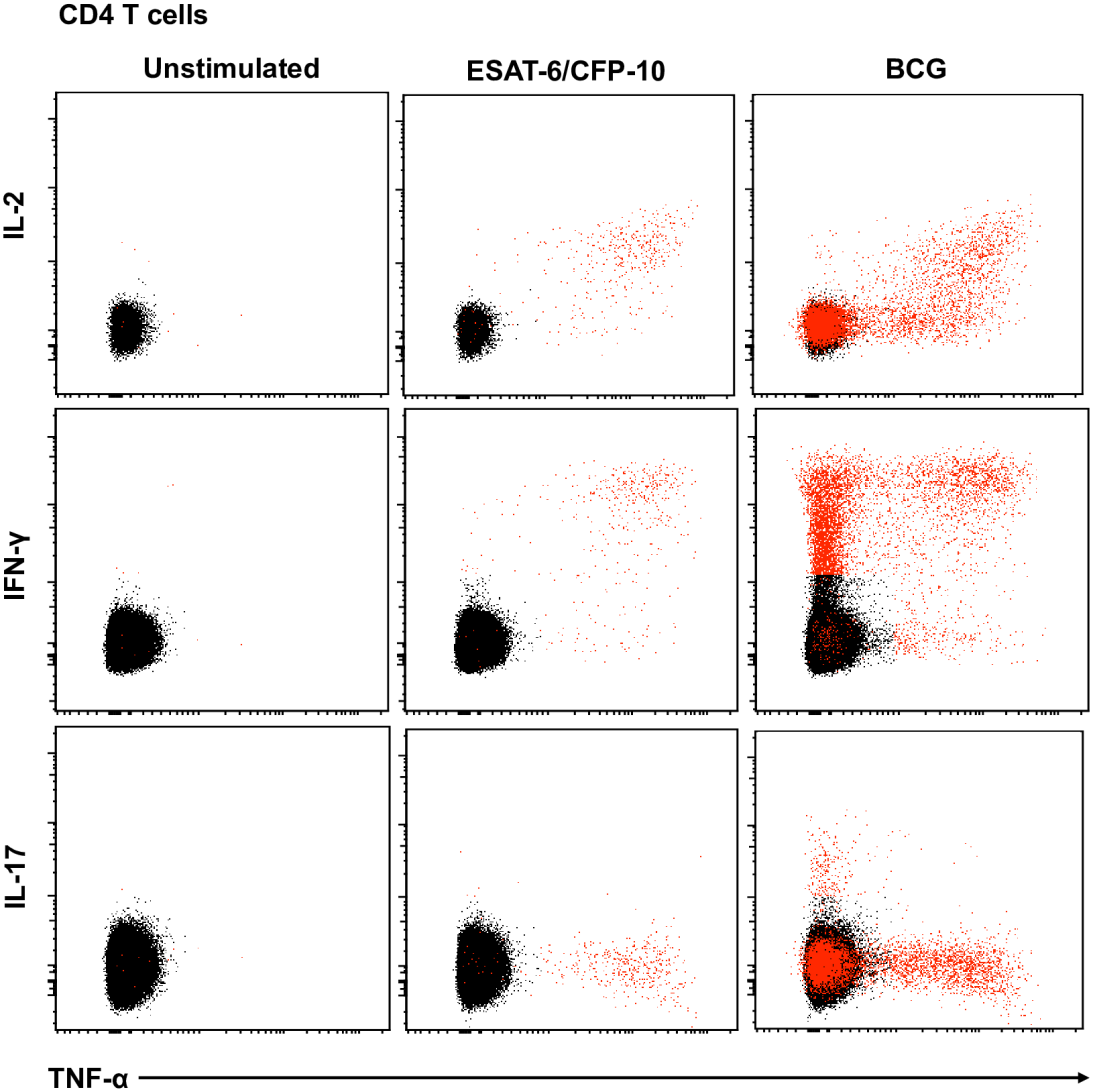
Representative flow cytometry plots of cytokine expression by CD4 T cells. CD4 T cells expressing IFN-γ, IL-2, TNF-α and/or IL-17 upon stimulation with BCG or ESAT-6/CFP-10 peptide pools for 12 hours, compared to an unstimulated control sample. The plots represent cytokine-positive cells (red) overlayed onto the background of the entire CD4 T cell parent population (black). Similar assessment was also performed for CD8 and γδ T cells (data not shown).

To explore if mycobacteria-specific CD4, CD8 or γδ T cell responses are associated with control of mycobacterial growth, whole blood from adults was stimulated with BCG or ESAT-6/CFP-10 peptide pools for 12 hours. Intracellular expression of IL-2, IFN-γ, TNF-α, and/or IL-17 was assessed by flow cytometry. The gating strategy and representative plots are shown in S2B Fig and Fig 5.

**Fig 6:**
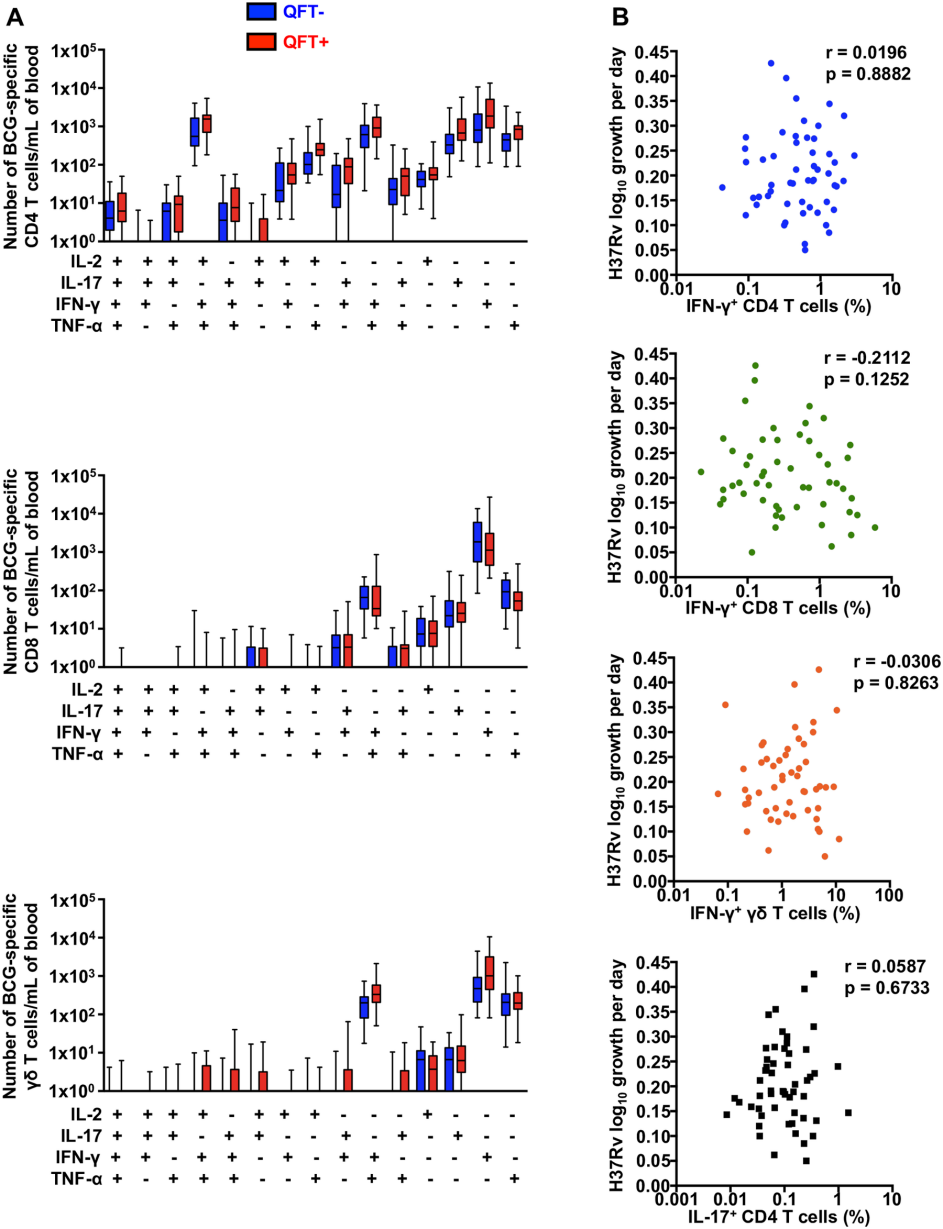
Mycobacteria-specific T cells in whole blood from adults and mycobacterial growth inhibition. (**A**) Absolute numbers of BCG-specific CD4, CD8 and γδ T cell subsets co-expressing IL-2, IFN-γ, TNF-α and/or IL-17, in whole bood from QFT+ (red) and QFT- (blue) adults. Medians are represented by the horizontal lines, interquartile ranges by the boxes, and ranges by the whiskers. The Mann-Whitney test was used to assess differences between QFT+ and QFT- adults and none were found to be different. (**B**) *M*.*tb* H37Rv growth plotted against frequencies of BCG-specific CD4, CD8 or γδ T cells expressing IFN-γ, or CD4 T cells expressing IL-17 in adults. R and p values were calculated using Spearman’s correlation analysis.

T cells are critical for control of *M*.*tb* growth in animal challenge models [33–38]. Because we did not observe differences in mycobacterial growth inhibition between *M*.*tb*-infected and uninfected individuals, we compared numbers of BCG-specific CD4, CD8 and γδ T cells between *M*.*tb*-infected and uninfected adults. None of the cytokine-expressing subsets of BCG-reactive CD4, CD8 and γδ T cells were found to be significantly different in abundance in blood from *M*.*tb*-infected and uninfected adults (Fig 6A).

BCG-specific CD4 T cells expressed IFN-γ alone or co-expressed IFN-γ with TNF and IL-2. We also detected an IL-17 expressing population of BCG-specific CD4 T cells (Fig 6B). By contrast, BCG-stimulated CD8 and γδ T cells predominantly expressed only IFN-γ.

Frequencies of BCG-specific CD4 T cells, CD8 T cells, or γδ T cells expressing the Th1 cytokine IFN-γ did not correlate with inhibition of *M*.*tb* H37Rv growth in whole blood (Fig 6B). Similarly, IL-17 expression by BCG-specific CD4 T cells also was not correlated with growth inhibition of *M*.*tb* H37Rv.

## Discussion

Twenty-three percent of the global population is currently infected with *M*.*tb*, yet only 3–10% of these individuals develop active TB disease within their lifetimes [1], providing compelling evidence that host immunity can successfully control bacterial growth [39]. This is further supported by a meta-analysis of 18 studies which estimated that individuals with latent *M*.*tb* infection had a 79% reduction in risk of TB disease after reinfection, than uninfected individuals [40]. It follows that *M*.*tb* infected persons must possess anti-mycobacterial immune responses that can control mycobacterial growth to a greater degree than uninfected persons. Consistent with this, studies from low TB burden settings showed that *in vitro* mycobacterial growth was significantly inhibited in whole blood from tuberculin-positive (*M*.*tb-*infected/previously exposed) individuals compared to tuberculin-negative individuals [17,19,20]. We determined whether *M*.*tb* infection would confer greater control of mycobacterial growth in persons from a high TB burden setting. Our results revealed that the capacity of whole blood to inhibit mycobacterial growth was not different between *M*.*tb*-infected and uninfected adults. Since prevalence of TB is typically about two-fold higher in males than females [2] we also investigated if gender was associated with *in vitro* mycobacterial growth, but observed no association. This finding was replicated in 2 other age groups comprising 8 year old children and 18 year old young adults.

The reasons for this result are not definitive but are likely important to understand. We showed that mycobacteria-specific T cell and innate cell responses were not significantly different between *M*.*tb*-infected and uninfected individuals, suggesting universally high immunological sensitization in the study population. Such high levels of immune responses to mycobacteria may derive from near universal BCG vaccination at birth, exposure to environmental mycobacteria and high levels of exposure to *M*.*tb* [30]. We postulate that this immunological sensitization may explain the equivalent growth inhibition observed in *M*.*tb*-infected and uninfected persons. A similar masking mechanism has been suggested to underlie the poor efficacy of BCG in settings with immunological sensitization to environmental mycobacteria [41].

We also observed no difference in mycobacterial growth inhibition in 8 year old children and young adults, despite the consistent epidemiological finding that pre-adolescent children above the age of 4 years are at significantly lower risk of TB compared with adolescents and adults [4,5]. This result may imply that factors other than immunity to mycobacteria underlie the age-associated risk of TB. However, in light of the broad dynamic range exhibited by the MGIA but limited ability of whole blood to restrict growth under these conditions, and the lack of differences between infected and uninfected persons, this interpretation is not strongly supported by our data. In fact, our finding that growth inhibition of *M*. *bovis* BCG and the *M*.*tb* strains H37Rv, HN878 and CDC1551, which exhibit a diverse range of virulence in animal models [31,32], correlated significantly, further questions the utility of the MGIA in our setting.

We report no association between IFN-γ QFT response and mycobacterial growth inhibition and did not identify any immunological outcomes that were associated with mycobacterial growth inhibition. This is consistent with findings from other human MGIA studies which reported no correlation between IFN-γ and mycobacterial growth inhibition [21,42,43]. IFN-γ is known to be essential for immunity against TB [44–46]. However, some murine studies also report no correlation between IFN-γ and protection against *M*.*tb* [10,11]. In other MGIA studies, IFN-γ was found to be essential in inhibiting mycobacterial growth in the murine model [23] and in the bovine model [24]. In addition to IFN-γ, previous studies have suggested an essential role for TNF-α [47–49] and IL-17 [50–52] in the anti-mycobacterial immune response. Vaccine induced protection in *M*.*tb*-infected mice correlated with IL-2 expressing CD4 T cells with a central memory phenotype [53,54].

However, depletion of CD4 and/or CD8 T cells in human [19,20], murine [55] and bovine [56] studies resulted in reduced ability to inhibit *M*.*tb* growth. Boom et al. also postulated that γδ T-cells may play a role in protective immunity against *M*.*tb* [57]. Studies by Worku and Hoft indicated that γδ T cells mediate superior levels of *M*.*tb* control [16,58].

To the best of our knowledge, this is the first time that the whole blood MGIA has been assessed in a setting with high burden of TB. Our results suggest that whole blood growth inhibition assays do not provide a useful measure of age-associated differential host control of *M*.*tb* infection in a high TB burden setting. We also found no innate or T cell correlates of control of mycobacerial growth. We propose that universally high levels of mycobacterial sensitization in persons from high TB burden settings may impart broad inhibition of mycobacterial growth, irrespective of *M*.*tb*-infection status. Such sensitization may occur through BCG vaccination given to nearly every South African infant at birth, although we cannot exclude possible exposure to environmental non-tuberculous mycobacteria. This is consistent with findings by Fletcher *et al*. who reported improved control of mycobacterial growth *in vitro*, following primary but not secondary vaccination with BCG [21]. Mycobacterial sensitization may thus mask the augmentative effects of mycobacterial sensitization on *M*.*tb* growth inhibition that is typical in low burden settings.

Our study has some limitations. The mycobacterial growth inhibition assay employed whole blood, yet observations of the periphery may not be representative of the lung, the predominant site of *M*.*tb* infection. Moreover, we could not measure all other potentially protective factors. For example, Worku and Hoft have previously reported that FAS, FAS ligand, perforin, granulysin and granzyme A were associated with mycobacterial growth inhibition [58]. In addition, haemoglobin (Hb) and iron levels present a potential confounding factor which could contribute to variability in mycobacterial growth when using whole blood. Tanner *et al*. reported an association between mean corpuscular haemoglobin (MCH) levels and *in vitro* BCG growth in whole blood from humans [59], an association that was not observed when using PBMC. In our study, participant MCH and Hb levels were not recorded and therefore we could not assess effects of MCH or Hb levels on mycobacterial growth. In addition, other factors have been shown to influence mycobacterial growth inhibition, including neutrophil counts [60], antibody-mediated responses [61,62] and complement [63], which may contribute to growth inhibition in whole blood assays but are not present in PBMC.

Finally, while MGIAs represent a level of systemic immune control of *M*.*tb* that is measurable in blood, it is clear that control of bacterial replication is determined at the level of individual granulomas and/or tissue sites of infection, which are typically highly heterogeneous [64–67] Regardless, a recent study by Fletcher *et al*. showed enhanced control of BCG using a PBMC-based MGIA 4–8 weeks after BCG vaccination in previously unsensitized individuals [21]. Efforts to further optimize mycobacterial growth inhibition assays and standardize experimental protocols between different laboratories have yielded promising results [68]. These data suggest that early prioritization of TB vaccine candidates for further clinical testing may be possible using blood-based MGIA assays in individuals selected with a lack of prior mycobacterial sensitization and from low TB burden settings.

## Supporting information

S1 Fig Lack of association between control of mycobacterial growth and *M*.*tb-*IFN-γ response. *M*.*tb* H37Rv growth was measured using whole blood MGIA and correlated against IFN-γ in supernatants from QuantiFERON-TB Gold In-Tube assay (QFT). P- and r- values were calculated using the Spearman rank correlation test. The dotted vertical line represents the QFT cut-off (0.35 IU/mL of IFN-γ) for diagnosis of *M*.*tb* infection.
(TIF)

S2 Fig Gating strategy intracellular cytokine staining assessment of innate cells and T cells by flow cytometry. (**A**) The innate cell gating strategy included 1) a time gate for consistency during acquisition; 2) a gate to exclude doublets; 3) a gate to exclude antibody aggregates; 4) neutrophils were gated on CD66^+^ population; and 5) CD66^-^ cells were further gated out to exclude T cells (CD3^+^), B cells (CD19^+^) and NK cells (CD335^+^), all on the same fluorochrome (BV421) used as a dump channel; 6) CD14^+^ monocytes and 7) CD14^-^CD11c^+^HLA-DR^+^ myeloid dendritic cells were identified from the remaining cells. (**B**) The T cell gating strategy included 1) a time gate for consistency during acquisition; 2) a gate to exclude antibody aggregates; 3) a lymphocyte gate based on expression of CD3^+^ T cells; 4) a gate to exclude doublets; 5) γδ T cells were identified based on expression of γδ-T cell receptor; the remaining cells were used to identify 6) and 7) CD4^-^CD8^+^ T cells, and 8) and 9) CD8^-^CD4^+^ T cells.
(TIF)
