# Supplementary figures and images for: Application of a whole blood mycobacterial growth inhibition assay to study immunity against Mycobacterium tuberculosis in a high tuberculosis burden population
Source: PLoS One. 2017 Sep 8;12(9):e0184563. doi: 10.1371/journal.pone.0184563 (PMC5590973; doi:10.1371/journal.pone.0184563)

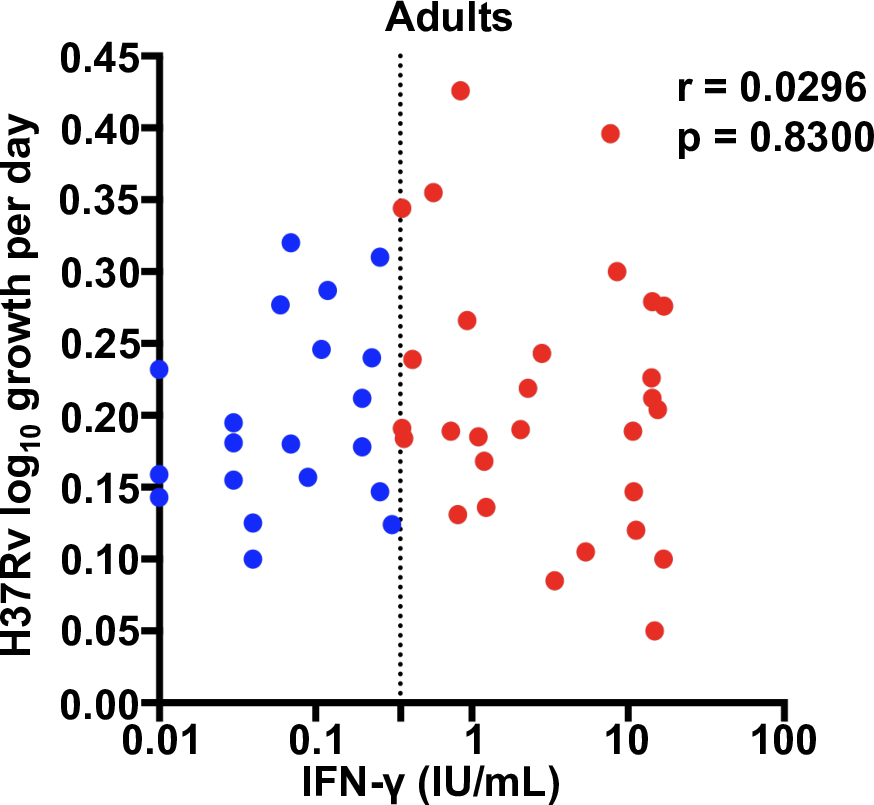

Supplement: S1 Fig — M.tb H37Rv growth was measured using whole blood MGIA and correlated against IFN-γ in supernatants from QuantiFERON-TB Gold In-Tube assay (QFT). P- and r- values were calculated using the Spearman rank correlation test. The dotted vertical line represents the QFT cut-off (0.35 IU/mL of IFN-γ) for diagnosis of M.tb infection. (TIF) [file pone.0184563.s001.tif]

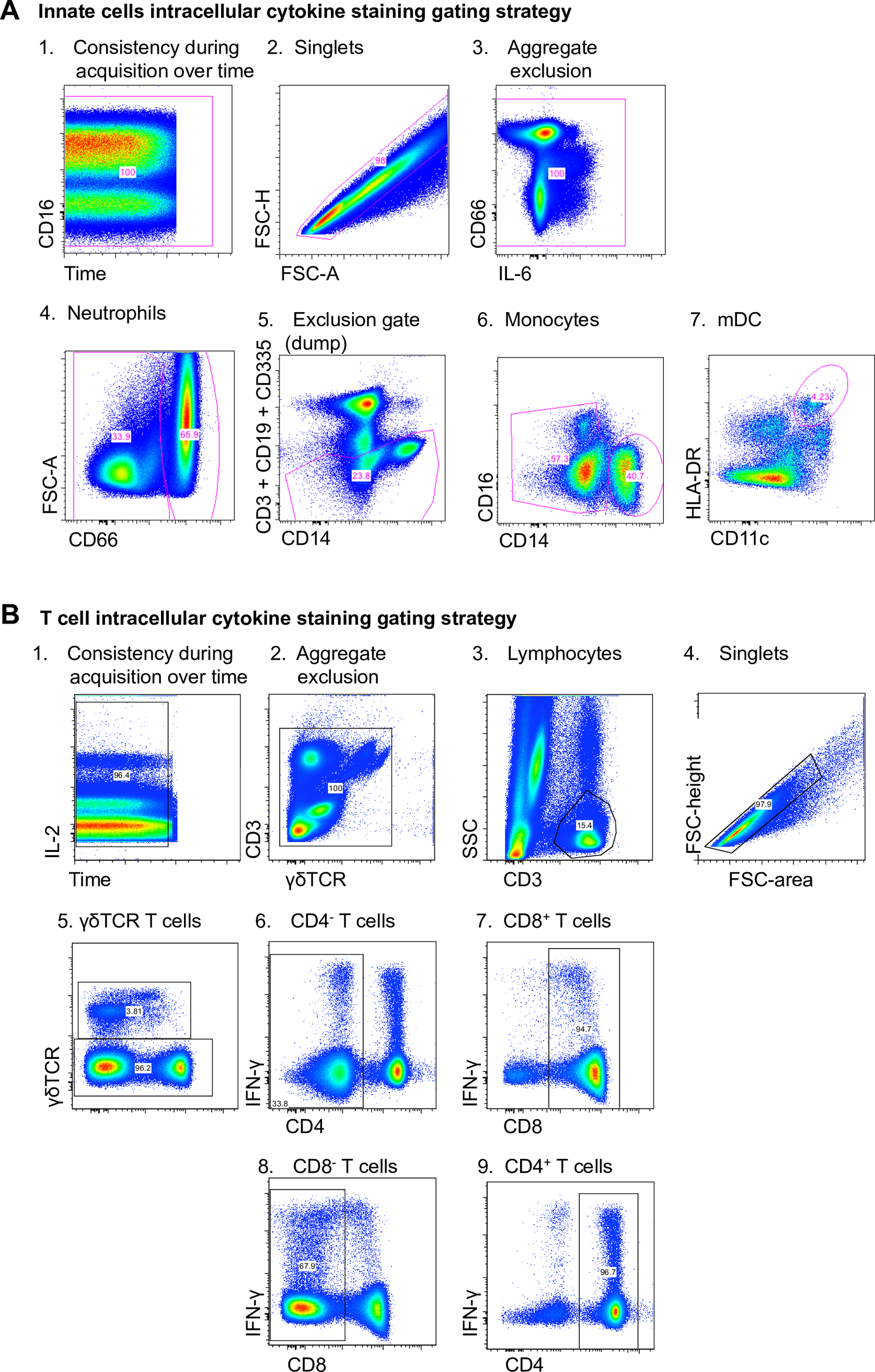

Supplement: S2 Fig — (A) The innate cell gating strategy included 1) a time gate for consistency during acquisition; 2) a gate to exclude doublets; 3) a gate to exclude antibody aggregates; 4) neutrophils were gated on CD66+ population; and 5) CD66- cells were further gated out to exclude T cells (CD3+), B cells (CD19+) and NK cells (CD335+), all on the same fluorochrome (BV421) used as a dump channel; 6) CD14+ monocytes and 7) CD14-CD11c+HLA-DR+ myeloid dendritic cells were identified from the remaining cells. (B) The T cell gating strategy included 1) a time gate for consistency during acquisition; 2) a gate to exclude antibody aggregates; 3) a lymphocyte gate based on expression of CD3+ T cells; 4) a gate to exclude doublets; 5) γδ T cells were identified based on expression of γδ-T cell receptor; the remaining cells were used to identify 6) and 7) CD4-CD8+ T cells, and 8) and 9) CD8-CD4+ T cells. (TIF) [file pone.0184563.s002.tif]
